# Installation of insecticide-treated durable wall lining: evaluation of attachment materials and product durability under field conditions

**DOI:** 10.1186/s13071-014-0508-4

**Published:** 2014-11-18

**Authors:** Louisa A Messenger, Marie Louise M Larsen, John H Thomas, Mark Rowland

**Affiliations:** Faculty of Infectious Tropical Diseases, London School of Hygiene and Tropical Medicine, London, UK; Technical University of Denmark (DTU), Lyngby, Denmark; Phoenix Ordinary LLC, Bridgewater, New Jersey USA

**Keywords:** Durable wall lining, Zerovector®, Malaria control, Installation, Feasibility, Nails and caps

## Abstract

**Background:**

Insecticide-treated durable wall lining (DL) is a new method of vector control designed to supplement LLINs and overcome two inherent limitations of LLINs and IRS: nightly behavioural compliance and short residual activity, respectively. DL is a deltamethrin-treated polyethylene material, which when used to cover interior house walls, functions as long-lasting IRS. Because the DL concept anticipates minimal upkeep, a primary challenge is how to guarantee correct household installation and *in situ* longevity for several years. Field trials were undertaken on various wall surfaces in Ghana to identify a logistically feasible, durable and re-usable method for DL wall attachment and to pilot new methods for assessing DL durability.

**Methods:**

Over fifty-five candidate attachment or fixing products, including mechanical fasteners, material anchors and adhesives, were evaluated for their ability to tolerate static loads (simulating long-term installation) and short-term heavy weights (imitating shock damage). Attachment products were also scored using qualitative logistical and feasibility criteria, including ease of preparation, grip of fixing to DL and possibility of re-use.

**Results:**

The stress tests provided a standardised, reproducible and reliable system for assessing fixing effectiveness and DL durability, with 64% (14/22) of adhesives and 15% (2/13) of mechanical fasteners failing to meet the minimum requirements of attaching DL to mud walls for set time periods. For most fixings, less outward load (0.2 – 8.0 kg) was required to detach DL from the wall, compared to downward load (0.2 – 19.2 kg). Fixings were better able to grip DL onto concrete than clay surfaces. Using a plastic nail cap to increase DL attachment area greatly improved grip and outward load tolerance, more so than varying nail size, length or texture.

**Conclusions:**

Based on a series of systematic stress tests, optimized fixing products for polyethylene DL wall attachment were identified. In parallel, a detailed and adaptable method of DL household installation was developed for routine deployment in malaria endemic areas. These standardized stress tests will form the basis for comparative evaluations of new types of DL textile, which incorporate non-pyrethroid insecticides to control malaria transmitted by resistant mosquito populations.

**Electronic supplementary material:**

The online version of this article (doi:10.1186/s13071-014-0508-4) contains supplementary material, which is available to authorized users.

## Background

In recent years, many African countries have implemented and rapidly scaled up indoor residual spraying (IRS) and long-lasting insecticidal nets (LLINs) as key components of malaria vector control [1-3]. While both interventions have considerably reduced disease morbidity when used alone [4,5] and in combination [6-8], problems of sustainability, increasing insecticide resistance and operational constraints now undermine movements towards malaria elimination [9-11]. In areas of stable or high transmission in sub-Saharan Africa, the infrastructure required to support large-scale, recurrent IRS campaigns of indefinite duration is not feasible [12]. Universal Coverage Campaigns (UCCs) of LLINs have significantly improved levels of household coverage, but often failed to achieve as great an impact on net usage [13,14].

Durable wall lining (ZeroVector® DL) may represent a potential alternate or complementary method of vector control to existing strategies. The current DL is a deltamethrin-treated high-density polyethylene (HDPE) material, which when used to cover interior house walls, functions as a long-lasting insecticidal reservoir. DL is designed to overcome some of the known limitations of conventional IRS and LLINs. Once installed, household protection is passive and not reliant on nightly behavioural compliance, unlike LLINs, and nor should it fall victim to householder or donor fatigue associated with annual rounds of spraying [15].

Preliminary field trials indicate DL will affect malaria transmission in a manner similar to IRS, by decreasing the density and longevity of indoor-resting vector populations [16-22]. The success of DL will therefore depend on executing and maintaining high community-level coverage over a period of years. Previous studies have already demonstrated that entomological efficacy and perceived aesthetic value are key determinants of DL user compliance [15,23,24]. However, the installation procedure is arguably the most crucial operational aspect to ensure long-term durability under field conditions and facilitate initial household acceptability. This situation is reminiscent of the evolution of LLIN technology. Initially, manufacturers and health authorities focused on developing an insecticidal product that would withstand repeated washing over three years [25]. Now that goal has been met, the graver concern, not yet achieved, is a netting durability longer than the one to three years of effective life of current materials [26].

This report describes the evaluation of candidate fixing products for ZeroVector® DL through several pilot field trials in rural Ghana. The aims of this study were: to identify a logistically feasible, durable and re-usable method for DL wall attachment; to establish an adaptable house installation protocol suitable for future community-level trials and routine deployment in malaria endemic areas; and to pilot standardised stress tests which could be used for comparative assessment of new types of fixings or the durability of novel DL materials.

## Methods

### Study site

**Figure 1:**
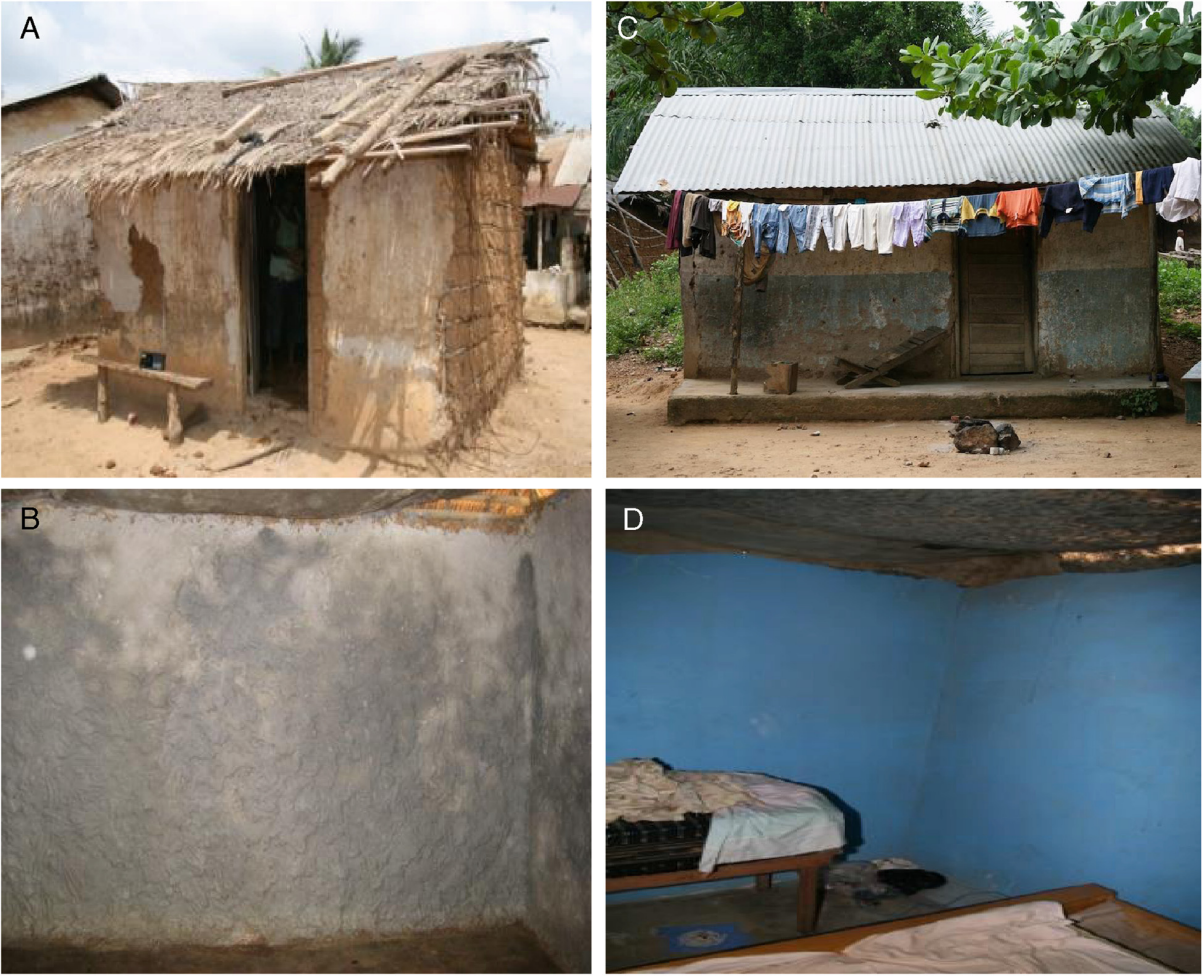
**Structural characteristics of phase 1 and 2 test houses. A**: Exterior of phase 1 test house constructed from mud, with internal clay covered walls. **B**: Interior of phase 1 test house. **C**: Exterior of phase 2 test house constructed from mud, with concrete covered walls. **D**: Interior of phase 2 test house.

In Anwona, Obuasi Municipality, Ashanti District, south-central Ghana (6°10’N, 1°43’06”W), the suitabilities of different fixing products for DL were assessed through two phases of stress tests, using village house walls that were representative of local construction materials. All phase 1 tests were performed in a house with clay covered mud walls (Figure 1A and B). Phase 2 evaluations were conducted using either clay or concrete rendered mud walls (Figure 1C and D).

### Durable wall lining material

**Figure 2:**
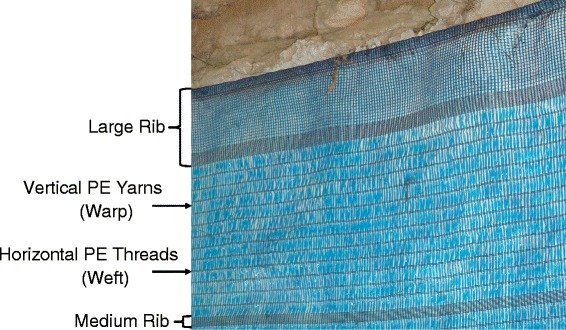
**Structure of DL material used to evaluate potential fixing products.**

The ZeroVector® DL evaluated during this trial was produced as large rolls (2.3 x 100 m) of blue 50% high-density polyethylene (HDPE) shade cloth (80 g/m^2^) with deltamethrin incorporated into the polymer during production (3.15 g/kg ± 25% *a.i.*) (supplied by Vestergaard Frandsen, Switzerland). The structure of the lining consisted of horizontal polyethylene threads (weft) knitted through vertical polyethylene yarns (warp) (Figure 2). Both outer edges of material were bordered by a finer threaded margin, or ‘large rib’, with two smaller ‘medium ribs’ in the middle of the fabric.

### Potential durable wall lining fixings

**Figure 3:**
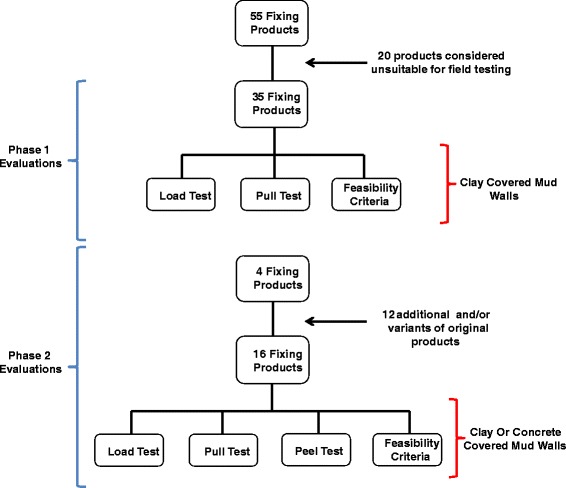
**Work scheme to evaluate potential DL fixing products.**

Fifty-five different fixings, including mechanical fasteners (nails, hooks, eyelets, staples and pins), material anchors (rope, cord and ties) and adhesives (tape, glue, putty and mud) were purchased from both local and international suppliers (Additional file 1: Table S1). Initially, twenty potential products were eliminated because they were considered unsuitable for mud walls. The remaining thirty-five fixings were evaluated under field conditions (Figure 3). Samples of DL were attached to test walls by potential products, to which constant or increasing loads of weight were applied to simulate real life scenarios within a controlled environment. Phase 2 evaluations were performed using a subset of the highest scoring fixings from phase 1.

### Experimental design: phase 1

#### Load test

**Figure 4:**
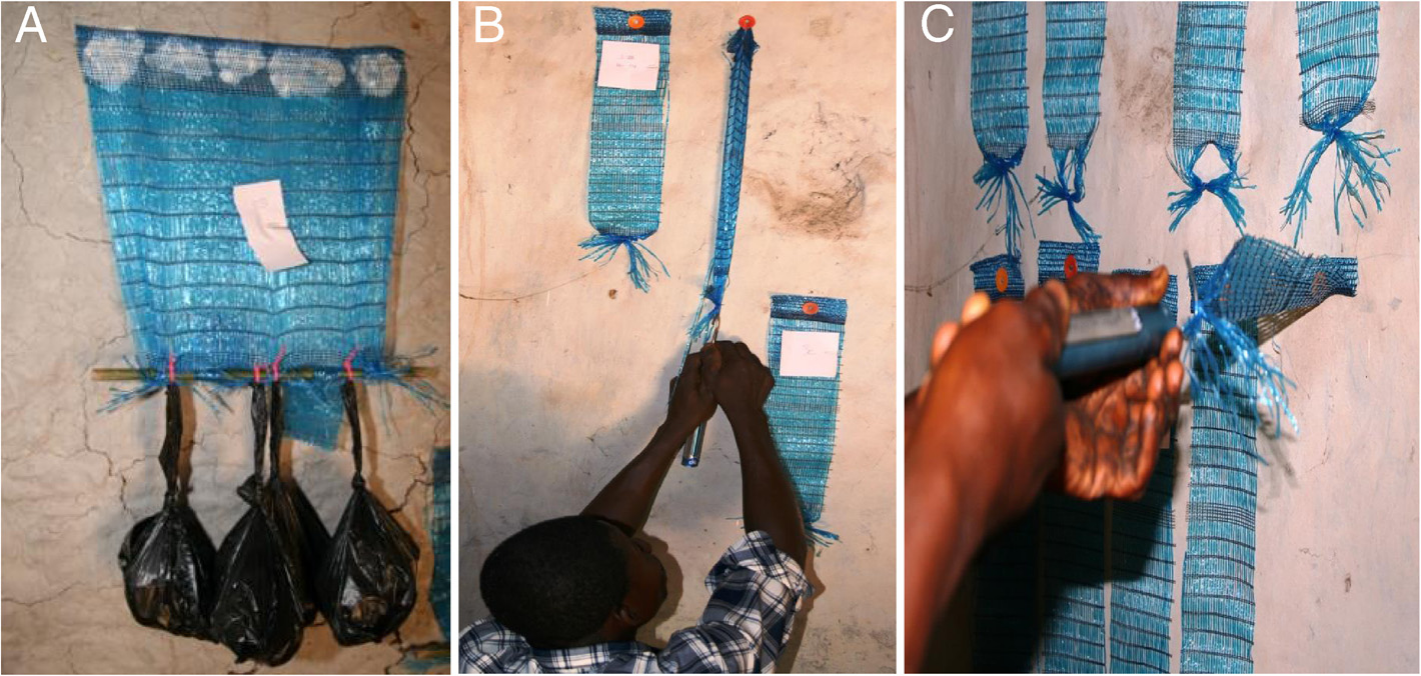
**Examples of** ***in situ*** **phase 1 and 2 stress tests. A**: Phase 1 load test evaluating AquaMend® underwater repair epoxy putty (product #43) with 4 kg at 112 hours. **B**: Phase 1 pull test evaluating Grip Rite® plastic cap with roofing nail (product #3). **C**: Phase 2 peel test evaluating Grip Rite® plastic cap with medium roofing nail (product #3b).

Static load was applied to the whole length of DL to imitate material attached to a wall for a number of years (‘load test’). DL samples measuring 30 × 30 cm were cut from one upper edge of the material roll and fixed to house walls with the large rib oriented towards the ceiling. To evaluate each potential mechanical fixing, four fasteners were used to attach a DL sample to the wall, positioned horizontally in the large rib at 2 cm, 10 cm, 20 cm, and 28 cm. Alternatively, other wall adhesives (tapes, glues, putty etc.) were applied to the whole area of the large rib (Figure 4A).

Following wall attachment, DL samples were left for 48 hours without adding weight. Initially 1 kg (±100 g) was applied over the whole length of each lining by suspending a plastic bag filled with rocks from the base of the textile, attached to a 30 cm bamboo pole. After 16 hours, three more plastic bags filled with rocks (1 kg ± 100 g/bag) were added (4 kg total weight). This weight (4 kg) was chosen to be considerably greater than anything expected under normal field conditions. The outcome of the load test was whether the DL was still attached to the wall after 112 hours.

#### Pull test

In a second test, increasing downward load was applied to the DL until the fixing product failed, to replicate a child pulling on the material unattended (‘pull test’). 10 × 40 cm DL samples (same orientation as the load test) were fixed with one mechanical item (positioned at 5 cm in the large rib) or an area of adhesive applied to the whole large rib. A handheld scale was attached to the base of the DL sample and pulled downwards at a 170° angle until either the fixing failed or the DL ripped (Figure 4B). The maximum weight required to pull the DL off the wall was recorded.

#### Additional feasibility criteria

Potential fixing products were also assessed using nine qualitative criteria: ease of preparation, ease of installation, grip at installation, grip with no load, grip of fixing to textile, aesthetics of material (with 4 kg load), possibility of re-using a failed fixing, impact of fixing failure on wall integrity and feasibility of fixing. For each criterion fixings were awarded an integer value between 1 and 10, with a higher number representing a more successful outcome, i.e. easier to install, better grip etc. In addition, each parameter was assigned a level of relative importance, with grip of fixing to textile and feasibility both considered to have the highest significance (1.0 for both). An overall score for a fixing product was given as the sum of its integers each multiplied by their respective relative importance values (possible maximum score of 58).

### Experimental design: phase 2

The top two mechanical and two adhesive fixing products in phase 1 were re-assessed in a second evaluation phase, along with 12 new products, which included variants of fixings from phase 1 (10 mechanical fixings and two glues; Additional file 2: Table S2); a single novel product (#67) was not tested during phase 1 due to logistical constraints. Phase 2 evaluated the performance of fixing products on two different wall substrates (clay or concrete) (Figure 1 and 3), using larger DL samples and with more limited attachment areas.

#### *Load test*

The load test was performed according to the same methodology as phase 1, over a longer time period, using larger areas of DL and heavier static load. 30 × 107 cm DL samples were cut as before, and fixed with two mechanical fasteners at 2 cm and 28 cm apart (adhesives were applied to two 4 cm areas at both ends of the large rib, separated by 22 cm). Following wall attachment, lining samples were left for 48 hours without adding weight. Subsequently, 6 kg (±600 g) was applied over the whole length of each DL sample, by suspending six plastic bags filled with rocks from the base of the lining (1 kg ± 100 g/bag), attached to a 30 cm bamboo pole. The outcome of the load test was whether the DL was still attached to the wall after 240 hours.

#### Pull test

As previously, in a second test increasing downward load was applied to the DL until the fixing product failed. 10 × 50 cm DL samples (same orientation as the load test) were fixed with one mechanical item (positioned at 5 cm in the large rib) or a 4 cm area of adhesive (applied to the centre of the large rib) and the maximum weight required to pull the DL off the wall was recorded.

#### Peel test

A third scenario was devised in which a DL sample was pulled horizontally outwards to assess how well the fixing product gripped the textile to the wall (‘peel test’). The rationale was that it requires less force to peel the DL from the wall than pulling it off downwards (in the ‘pull test’). To measure this, 10 × 50 cm samples of DL (same orientation as other tests) were fixed with one mechanical item (positioned at 5 cm in the large rib) or a 4 cm area of adhesive (applied to the centre of the large rib). A handheld scale was attached to the base of the DL sample and pulled outwards at a 90° angle until the fixing failed (Figure 4C). The maximum amount of outward load required to detach the DL from the wall was recorded.

#### Additional feasibility criteria

Potential fixing products were ranked using the same nine qualitative criteria from phase 1 and scoring methodology.

### Household installation of durable wall lining

Following the evaluation of fixings, a detailed method of DL household installation was developed using the final choice of fixing product.

## Results

### Initial choice of fixings

A total of 55 fixing products were purchased both locally and from international suppliers. Twenty potential fixings were eliminated prior to field evaluation because they were deemed unfeasible for wall attachment. These included all material anchors (#45–55), as well as drawing pins (#5), concrete nails (#15), screw eyelets (#17 and 18), hooks (#19), wood fasteners (#20) and cable plastic staples (#22) (Additional file 1: Table S1).

### Phase 1: load test

Thirty-five potential fixing products (13 mechanical fasteners, 12 glues and 10 tapes) were evaluated for their ability to tolerate moderate static load. Within the initial 48 hours of wall attachment without weight, six fixings fell down (five tapes; #23, 27, 28, 29, 31 and one glue; #34). Two additional products failed while hanging the first kilo of weight (one mechanical fastener; #21 and one tape; #32). After 112 hours, eight more potential fixings had fallen down (one mechanical fastener; #9, four tapes; #24, 25, 26, 30, and three glues; #35, 42 and 44). Overall 54% (19/35) of fixings could withstand a 4 kg load for 112 hours, representing 85% (11/13) of mechanical products and 67% (8/12) of glues (Table 1). All ten potential tapes failed the load test.

**Table 1:** **Phase 1 load and pull test results for 33 potential durable wall lining fixing products**

**Type of fixing**	**Product #**	**Product**	**Pull test: Maximum load before fixing failure (kg)**	**Load test: Fixing attached to wall with 4 kg at 112 hrs**
Glue	37	Liquid Nails®*	17.6	Yes
Mechanical	1	Steel Hooks	16.6	Yes
Mechanical	9	Metal Staples	12.5	No
Mechanical	13	Nails	11.5	Yes
Glue	33	Fortissimo MS Glue	11.5	Yes
Glue	38	DAP® Weldwood® Contact Cement	11.5	Yes
Mechanical	2	Steel Hooks	10.6	Yes
Mechanical	3	Roofing Nail With Grip Rite® Plastic Cap*	10.6	Yes
Glue	35	Durabond	10.5	No
Mechanical	6	Fasteners	9.5	Yes
Mechanical	7	Cardboard Nails	8.6	Yes
Mechanical	4	Upholster Nails	8.2	Yes
Glue	43	AquaMend® Underwater Repair Epoxy Putty	8.2	Yes
Mechanical	8	Metal Staples*	8.0	Yes
Glue	41	TEKNAbond® Multi-Purpose Wall Size/Adhesive	7.5	Yes
Tape	24	Double-Sided Carpet Tape	7.4	No
Glue	39	Stick-Ease Wall Covering Seam Repair	7.2	Yes
Tape	30	Double-Sided Tape	6.4	No
Glue	36	PowerGrab*	6.2	Yes
Tape	26	Outdoor Mounting Tape	6.0	No
Glue	42	DAP® Blue STIK™ Reusable Adhesive Putty	5.5	No
Mechanical	10	Metal Staples	5.0	Yes
Mechanical	21	Non-Metallic Cable Plastic Staples	4.8	No
Mechanical	16	Steel Nails	4.5	Yes
Tape	29	Double-Sided Tape	4.4	No
Mechanical	11	Metal Staples	3.8	Yes
Glue	44	Mud	3.0	No
Tape	25	Outdoor Mounting Tape	2.8	No
Tape	27	PowerGrab Heavy Duty Adhesive	2.5	No
Glue	40	Border Paste	2.5	Yes
Tape	23	Double-Sided Carpet Tape	0	No
Tape	28	Double-Sided Universal Tape	0	No
Tape	31	Double-Sided Tape	0	No
Tape	32	Power Tape	0	No
Glue	34	Ponal Paper Glue	0	No

*indicates fixing products which were tested in Phase 2.

Products are ordered according to their performance during the pull test.

### Phase 1: pull test

For each potential fixing, the maximum weight required to detach the DL from the wall was measured. The amount of load sustained by fixing products ranged from 2.5 - 17.6 kg (Table 1). Five products could not tolerate any weight during the pull test (four tapes; #23, 28, 31 and 32 and one glue; #34). Fourteen fixings could withstand more than 8 kg (twice the weight of the load test): nine mechanical fasteners (#1, 2, 3, 4, 6, 7, 8, 9 and 13) and five glues (#33, 35, 37, 38 and 43). While the data for pull and load test were broadly correlated (*p* = 0.0014, Wilcoxon rank sum test), for eight products, results were discordant. In these cases, fixings that failed the 4 kg load test, were able to tolerate in excess of this weight during the pull test, e.g. metal staples (#9), which required 12.5 kg to detach from the wall, had failed by 112 hours with 4 kg of sustained weight.

### Phase 1: additional feasibility criteria

All fixing products were scored according to nine qualitative criteria which considered logistical and feasibility aspects, including preparation complexity, installation time, number of tools required, reusability of failed fixings and aesthetics of attached DL. Table 2 ranks the top 10 phase 1 fixing products according to their feasibility scores.

**Table 2:** **Phase 1 top 10 potential durable wall lining fixing products ranked by feasibility criteria**

**Type of fixing**	**Product #**	**Product**	**Ease of preparation**		**Ease of installation**		**Grip at installation**		**Grip with no load**		**Grip of product**		**Aesthetics of fabric (4 kg Load)**		**Re-use of failed fixing**		**Failure of fixing on wall**		**Feasibility**		**Overall score**
				0.4**		**0.7**		**0.8**		**1.0**		**1.0**		**0.4**		**0.3**		**0.2**		**1.0**	
Mechanical	3	Roofing Nail With Grip Rite® Plastic Cap*	10.0	4.0	9.5	6.65	9.0	7.2	10.0	10.0	7.0	7.0	7.0	2.8	10.0	3.0	7.0	1.4	8.5	8.5	**50.55**
Mechanical	8	Metal Staples*	10.0	4.0	9.0	6.3	9.0	7.2	10.0	10.0	8.0	8.0	6.0	2.4	10.0	3.0	8.0	1.6	7.5	7.5	**50.0**
Mechanical	9	Metal Staples	10.0	4.0	8.5	5.95	8.5	6.8	10.0	10.0	7.8	7.8	4.0	1.6	10.0	3.0	8.0	1.6	7.0	7.0	47.75
Tape	26	Outdoor Mounting Tape	5.0	2.0	9.0	6.3	7.5	6.0	10.0	10.0	10.0	10.0	10.0	4.0	0	0	10.0	2.0	7.0	7.0	47.3
Mechanical	6	Fasteners	10.0	4.0	9.0	6.3	6.0	4.8	10.0	10.0	6.5	6.5	5.5	2.2	10.0	3.0	8.0	1.6	7.5	7.5	45.9
Mechanical	16	Steel Nails	10.0	4.0	10.0	7.0	8.0	6.4	10.0	10.0	5.5	5.5	6.0	2.4	10.0	3.0	5.0	1.0	6.5	6.5	45.8
Mechanical	13	Nails	10.0	4.0	8.5	5.95	9.0	7.2	10.0	10.0	5.0	5.0	6.0	2.4	10.0	3.0	6.0	1.2	6.5	6.5	45.25
Glue	42	DAP® Blue STIK^TM^ reusable adhesive putty	6.0	2.4	8.5	5.95	7.5	6.0	10.0	10.0	8.5	8.5	8.5	3.4	8.0	2.4	8.0	1.6	5.0	5.0	45.25
Glue	37	Liquid Nails®*	3.5	1.4	6.0	4.2	7.0	5.6	10.0	10.0	10.0	10.0	10.0	4.0	0	0	9.0	1.8	7.0	7.0	**44.0**
Glue	36	PowerGrab Heavy Duty Adhesive*	5.0	2.0	6.0	4.2	7.0	5.6	10.0	10.0	10.0	10.0	10.0	4.0	0	0	9.5	1.9	6.0	6.0	**43.7**

*indicates fixing products which were tested in Phase 2; overall feasibility scores are highlighted in bold.

**indicates relative importance value.

Potential adhesives generally scored the lowest for ease of preparation and installation and possibility of re-use but amongst the highest for grip with no load, grip of fixing to textile and aesthetics of textile with 4 kg. Mechanical fixings, while ranked favourably for ease of preparation, grip with no load and possibility of re-use, were rated poorly for impact of fixing failure on wall integrity, aesthetics of fabric with 4 kg and grip of fixing to textile.

After phase 1 of testing, all fixings with values under 40 were excluded from further evaluation, including 38% (5/13) of mechanical fasteners, 80% (8/10) of tapes and 67% (8/12) of glues. From the remaining 14 products, the top two mechanical fixings (roofing nail with Grip Rite® plastic cap #3 and metal staples #8) and glues (PowerGrab Heavy Duty Adhesive #36 and Liquid Nails® #37), based on combined load test, pull test and feasibility scores, were chosen for phase 2 evaluations (Figure 3).

### Phase 2: load test

Sixteen potential fixing products (12 mechanical fasteners and four glues) were evaluated on two different wall substrates in the phase 2 load test (Table 3). Nine of the mechanical fasteners consisted of combinations of different nail lengths (2.54, 3.8 or 4.5 cm roofing nails), shaft textures (smooth, fluted, spiral or marked) and plastic nail caps (Grip Rite® or Bostitch®).

**Table 3:** **Phase 2 load, pull and peel test results for 16 potential durable wall lining fixing products**

**Type of fixing**	**Product #**	**Product**	**Peel test on clay: Maximum load before fixing failure (kg)**	**Peel test on concrete: Maximum load before fixing failure (kg)**	**Pull test on clay: Maximum load before fixing failure (kg)**	**Pull test on concrete: Maximum load before fixing failure (kg)**	**Load test on clay: Fixing attached to wall with 6 kg at 240 hrs**	**Load test on concrete: Fixing attached to wall with 6 kg at 240 hrs**
Mechanical	56	Marked nail with Bostitch® plastic cap	8.0	12.0	19.2	18.6	Yes	Yes
Mechanical	59	Spiral shank nail with Bostitch® plastic cap	5.6	6.2	17.4	17.0	Yes	Yes
Mechanical	8	Metal staples (edged, large)	4.0	3.0	9.8	14.8	Yes	Yes
Mechanical	60	Marked nail	4.0	2.4	6.0	1.4	No	Yes
Mechanical	62	Spiral shank nail #59 with Grip Rite® plastic cap	3.4	5.0	19.0	17.2	Yes	Yes
Mechanical	3c	Roofing nail (large) with Grip Rite® plastic cap	3.0	14.6	4.0	18.2	Yes	Yes
Mechanical	3b	Roofing nail (medium) with Grip Rite® plastic cap	3.0	5.2	18.5	18.6	Yes	Yes
Mechanical	63	Roofing nail from #3b with Bostitch® plastic cap	3.0	5.2	19.0	18.6	Yes	Yes
Mechanical	61	Roofing nail #3 with Bostitch® plastic cap	2.0	3.2	19.0	16.2	Yes	Yes
Mechanical	58	Fasteners	1.8	0	7.1	0	Yes	Yes
Mechanical	57	Fluted shank masonry with Bostitch® plastic cap	1.6	3.0	3.6	6.8	Yes	No
Glue	67	3 M Spray	0.8	1.0	4.8	3.5	No	No
Mechanical	3	Roofing nail (small) with Grip Rite® plastic cap	0.2	0.4	15.8	18.6	Yes	Yes
Glue	36	PowerGrab	0	1.8	0.2	9.4	No	No
Glue	37	Liquid Nails® (LN-700)	0	1.2	3.8	4.5	No	No
Glue	68	Liquid Nails® (LN-701)	0	0.8	0.2	5.0	No	No

Products are ranked according to their performance during the peel test on clay.

Overall 83% (10/12) of mechanical fixings were able to withstand 6 kg of load for 240 hours on clay and concrete walls (marked nail #60 failed on clay and fluted shank masonry with Bostitch® plastic cap #57 detached from concrete after 72 hours). All four potential glue products failed the load test on both types of wall by 96 hours.

### Phase 2: pull test

In the phase 2 pull tests, the amount of load sustained by fixing products ranged from 0.2 - 19.2 kg and from 1.4 - 18.6 kg on clay and concrete walls, respectively (Table 3). One mechanical fixing could not tolerate any downward weight on concrete (fasteners #58) but sustained 7.1 kg on clay before failure. There was no clear grip superiority of fixings according to wall substrate; 50% (8/16) of products were able to withstand more downward load on clay compared to concrete walls. The average difference in weight between pull tests on clay and concrete was 3.7 kg (±3.8; range of 0.1 - 14.2 kg).

There was no apparent grip advantage between types of nail cap (18.5 *vs.* 19 kg on clay and 18.6 *vs.* 18.6 kg on concrete for 3.8 cm nail with Grip Rite® and Bostitch® caps, respectively) or nail length (19 *vs.* 19 kg on clay and 16.2 *vs.* 18.6 kg on concrete for 2.54 and 3.8 cm roofing nails with Bostitch® caps, respectively). Nail texture also did not alter pull test performance (19 *vs.* 19.2 *vs.* 17.4 kg on clay for smooth 3.8 cm roofing nail (#63), marked nail (#56) and spiral nail (#59), all with Bostitch® caps), with the exception of fluted nail with Bostitch® cap (#57), which tolerated the lowest amount of downward weight out of all potential nail fixings on both types of wall (3.6 and 6.8 kg on clay and concrete, respectively). However, nail caps greatly increased the amount of load required to detach DL, e.g. marked nail (#60) alone sustained 6 kg *vs.* 19.2 kg on clay when combined with a Bostitch® cap (#56).

Results for the load and pull tests were consistent for the majority of fixings. Three products, which failed the load test, were able to withstand 6 kg or more in one of the pull tests (Powergrad #36 failed after 9.4 kg on concrete, fluted shank masonry with Bostitch® plastic cap #57 after 6.8 kg on concrete and marked nail #60 after 6 kg on clay).

### Phase 2: peel test

The amount of maximum outward weight required to detach DL ranged from 0.2 - 8 kg and 0.4 - 14.6 kg on clay and concrete walls, respectively (Table 3). Three glues failed the peel test on clay (#36, 37 and 68) and one mechanical fixing could not tolerate any load on concrete walls (fasteners #58). Eighty-one per cent (13/16) of potential products were able to withstand more outward load on concrete compared to clay. The average difference in weight between peel tests on different wall substrates was 2.1 kg (±2.7, range of 0.2 - 11.6 kg). One product (large roofing nail with Grip Rite® plastic cap #3c) produced the largest discrepant results between wall substrates in both pull and peel tests (3 kg and 4 kg on clay and 14.6 kg and 18.2 kg on concrete for peel and pull tests, respectively).

Similar to the pull test results, the presence of a nail cap greatly increased tolerance for outward weight (4 *vs.* 8 kg on clay and 2.4 *vs.* 12 kg on concrete for marked nail #60 and marked nail with Bostitch® cap #56, respectively). Likewise there was no difference between type of nail caps (3 kg on clay and 5.2 kg on concrete for 3.8 cm nail with Grip Rite® and Bostitch® caps, respectively) or nail length (2 *vs.* 3 kg on clay and 3.2 *vs.* 5.2 kg on concrete for Bostitch® caps with 2.54 and 3.8 cm roofing nails, respectively). Regarding nail texture, marked nails were able to withstand more outward load (8 and 12 kg on clay and concrete, respectively) compared to smooth roofing nails (3 and 5.2 kg on clay and concrete, respectively) or spiral nails (5.6 and 6.2 kg on clay and concrete, respectively) (all with Bostitch® caps).

The majority (94%; 15/16) of fixing products tolerated less outward weight compared to downward load (marked nail #60 could withstand 1.4 *vs.* 2.4 kg on concrete pull and peel tests, respectively).

### Phase 2: additional feasibility criteria

Consistent with phase 1 results, the four potential glues scored the lowest for ease of preparation and installation and overall feasibility, but ranked highly for aesthetics of textile with load and grip at installation.

While all nail fixings performed well for ease of preparation and installation, some differences emerged with respect to the two types of nail caps. During the pull and peel tests it became apparent that the Grip Rite® cap was brittle and easily fractured around the nail head or along the entire cap length. By contrast, the Bostitch® cap would bend but ultimately fail because of insufficient nail grip. Considering the Grip Rite® cap was essentially unusable following fixing failure all four mechanical fixings with this type of cap were excluded from further analysis (#3, 3b, 3c and 62).

Other mechanical fixings, such as metal staples, while ranked among the highest for the peel test (4 and 3 kg on clay and concrete, respectively), scored the lowest for ease of installation, grip of fixing to textile, aesthetics of textile with load and overall feasibility and were also eliminated at this stage.

The top three potential fixings, based on combined load, pull and peel tests and qualitative scores, were marked nail with Bostitch® cap (#56), spiral nail with Bostitch® cap (#59) and 3.8 cm roofing nail with Bostitch® cap (#63).

### Household installation of durable wall lining

A standardized, detailed DL installation procedure, using Bostitch® plastic caps with locally-sourced steel nails, was devised for prospective community-level trials. This protocol was designed to be reproducible in areas of high heterogeneity among household structures, to maximize time and resources, minimize material wastage and provide suggestions for local installation adaptation, previously encountered during pilot field studies [15,23,24]. A full description is given in Additional file 3: File S1.

## Discussion

Insecticide-treated durable wall lining is a new vector control strategy developed to overcome two of the main drawbacks of LLINs and IRS, the dependence on behavioural compliance and short residual activity of insecticide, respectively. It may represent a viable alternative to conventional methods or an addendum to concurrent vector control campaigns in epidemiological situations where disease control has plateaued.

Because DL is a long-lasting passive intervention that should require minimal maintenance, one of its primary operational challenges remains how to guarantee *in situ* longevity and correct household installation over a number of years, without external interference. Ideally, a candidate DL fixing would satisfy the following criteria: (1) demonstrates long-term durability and the potential to withstand temporary high impact damage; (2) is reusable or easily replaceable by the house owner, in case of repairs; (3) attaches DL evenly, ensuring a uniform surface of available insecticide; and (4) is logistically feasible for widespread application at the community-level.

This study evaluated the suitability of different fixing products for DL wall attachment, using stress tests designed to replicate gradual damage experienced in a household setting. Initially 55 fixing products were assessed for their ability to tolerate moderate load (imitating long-term installation) and the effect of short-term heavy weight (to replicate the actions of a child). Early into the phase 1 evaluations it became evident that adhesive tapes were impractical and did not warrant further consideration; all of them failed the load test, most were unable to withstand any downward weight, they were complex to prepare and not re-usable. Instead the majority of the highest scoring products during phase 1 were mechanical fixings, principally steel nails and large staples. Amongst the mechanical products differences emerged regarding head-length ratios. Longer nails and staples were unable to attach completely into the wall, fixing DL unevenly, while shorter ones or those with larger heads relative to their length were more difficult to maneuver during installation. Interestingly, the grips of some adhesive glues were equivalent or exceeded those of the strongest mechanical products. However, glues also suffer from some intrinsic limitations: the amount used per DL is difficult to standardize, they are time consuming to install in terms of drying time, not re-usable, prone to wastage and, if incorrectly applied, can actively block areas of insecticide.

Based on the feasibility criteria, potential fixing products fell into three broad categories, those that were simple to prepare and install (mechanical), those that gripped the DL and fixed it uniformly (adhesives) and those that were reusable upon failure (mechanical). At this stage preference was given to products that were logistically easier to implement with the aim of reducing the most laborious and time-consuming aspects of the installation process.

The phase 2 evaluations focused on a subset of the strongest mechanical and adhesive glue products from phase 1, using two different types of common house walls. In addition, a third stress test was introduced to measure how well potential products gripped DL directly onto the wall surface. For almost all fixings, less outward weight was required to detach DL from the wall and fixings were better able to grip DL onto concrete than clay surfaces. Results from both pull and peel tests indicated that using a plastic nail cap to increase DL attachment area greatly improved grip and outward load tolerance, more so than variation in nail size, length or texture. While there was no difference in weight performance between the two types of potential plastic caps, the Bostitch® cap was deemed superior because it was reusable upon fixing failure. The final choice of nail to use in conjunction with Bostitch® caps will likely be determined by local availability, predominant housing substrate within the community and cost-effectiveness.

Following the evaluation of fixings, a reproducible DL installation protocol was developed for forthcoming community-level trials (Additional file 3: File S1). This protocol serves as a technical guideline for the installation of DL by National Malaria Control Programmes (NMCPs) and Non-Governmental Organizations (NGOs).

How DL will be installed as a matter of routine is not yet established. Will DL become the new long-lasting alternative to IRS for NMCPs, which can incorporate insecticides unsuitable for use on LLINs? Will it form part of the movement lobbying for house improvements as a new precedent in vector control [27,28]? Or, at the other extreme, will it merely serve as a niche product supplied by wealthy corporations to protect their mining or plantation communities? It is too early to tell. First it will be necessary to perform a community-randomized trial to demonstrate proof of concept for malaria control, as was conducted for insecticide-impregnated plastic sheeting used by refugees and other populations displaced by war or natural disasters [21]. The initials results are promising. Prolonged residual control of mosquitoes has been confirmed in household evaluations of DL in several African countries [23]. Recipients in rural areas, most at risk of malaria, readily accepted the intervention declaring it protected them and improved their houses aesthetically [15]. Finally, this third field trial, with its focus on mechanisms of DL attachment and methods for assessing strength and grip, has demonstrated that durability of installation is possible in practice.

The DL currently in use is based on polyethylene shade cloth impregnated with deltamethrin. New types of DL incorporating a range of insecticides will be required in the future to improve control of the increasing number of vector populations that are showing resistance to pyrethroids. The new DLs will likely be made from a variety of different textiles and may utilize alternative fixings in addition to the ones tested herein. The stress test methods piloted for polyethylene DL can form the basis for guidelines to assess any new candidate fixing product in laboratory testing or small-scale trials of DL durability in selected households or experimental huts [29,30]. In addition to the mode of attachment, the relative strength and durability of the textile itself will need to be evaluated against the current gold standard polyethylene shade cloth. Once these products are more established, the longer term integrity and durability of DL under field conditions, including its capacity to withstand the wear and tear of household use, will need to be the subject of WHO Pesticide Evaluation Scheme guidelines to enable novel products to be assessed in a regulated and standardized manner.

### Study limitations

While this preliminary study is important to establish parameters and a methodology to assess candidate DL fixing products and durability, there are several limitations with experimental design that should be considered. This study only measured short-term durability of potential fixings for up to 240 hours in the phase 2 tests. To date, the longest field evaluation of DL durability among houses constructed from wood, mud or concrete, reported less than one quarter experienced failed nails with plastic caps over 12 months [15,23]. Longitudinal studies of DL, including detailed observations of fixings and material deterioration and the extent of maintenance by householders, are required to relate DL longevity under field conditions with the stress tests performed herein and to improve the overall installation protocol.

Two types of wall substrate were chosen for evaluation (clay and concrete covered mud walls) based on their abundance in local villages. It might be anticipated that certain fixing products would perform even poorer on mud walls with no surface rendering. Previous pilot studies have also described problems fixing nails into rough wooden surfaces, such as house beams [24]. As DL is executed among a wider range of household structures, it is likely that more local fixing product adaptations will be developed on a country-by-country basis.

Lastly, the qualitative feasibility criteria used to measure aesthetic and logistical aspects of potential fixing products were largely subjective. These parameters were only scored by one experimenter and future studies should incorporate independent validation by multiple assessors.

## Conclusions

One of the primary operational challenges associated with durable wall lining is how to maintain *in situ* durability and correct household installation over a period of years. Following a series of systematic stress tests, recommendations are presented for a logistically feasible, durable and re-usable method for DL wall attachment. A fixing evaluation methodology is described which can be used to assess novel candidate products and new DL textiles in the laboratory and field. An adaptable installation protocol was also developed for routine use in community-level trials or by malaria control programmes.

## Additional files

Additional file 1:
**Table S1.** Specifications of fifty-five potential durable wall lining fixing products evaluated during phase 1. Additional file 2:
**Table S2.** Specifications of sixteen potential durable wall lining fixing products evaluated during phase 2. Additional file 3:
**File S1.** Durable wall lining household installation protocol.
