# Supplementary material for: Installation of insecticide-treated durable wall lining: evaluation of attachment materials and product durability under field conditions
Source: Parasit Vectors. 2014 Nov 18;7:508. doi: 10.1186/s13071-014-0508-4 (PMC4246572; doi:10.1186/s13071-014-0508-4)
Supplement: Additional file 1: — Table S1. Specifications of fifty-five potential durable wall lining fixing products evaluated during phase 1. [file 13071_2014_508_MOESM1_ESM.docx]

Supplementary Table 1. Specifications of fifty-five potential durable wall lining fixing products evaluated during phase 1.

| **Type of fixing** | **Product Number #** | **Product** | **Product Image** | **Manufacturer, Source** | **Description** | **Size** | **Field Evaluation** |
| --- | --- | --- | --- | --- | --- | --- | --- |
| Mechanical | 1 | Steel hooks (large) | 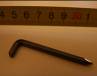 | Silvan, Denmark | Blue, tempered | 0.4 x 6 cm | Yes |
|  | 2 | Steel hooks (small) | 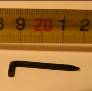 | Silvan, Denmark | Blue, tempered | 0.2 x 3 cm | Yes |
|  | 3 | Roofing nail with Grip Rite^®^ plastic cap | 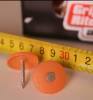 | Grip Rite Prime Source^®^ Building products Inc., TX, USA | Stainless steel nail with orange plastic cap covering nail head | 0.2 x 2.54 cm  (2.4 cm cap diameter) | Yes |
|  | 4 | Upholster nails | 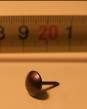 | Silvan, Denmark | Browned nail with rounded head | 1 cm | Yes |
|  | 5 | Drawing pins | 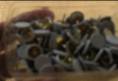 | Føtex, Denmark | Flat nail head with plastic coating | 0.9 cm | No |
|  | 6 | Fasteners | 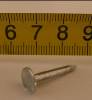 | Grip Rite Prime Source^®^ Building Products Inc., TX, USA | Electrogalvanized roofing nail | 0.30 x 2.2 cm | Yes |
|  | 7 | Cardboard nails | 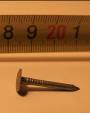 | Silvan, Denmark | Zinc coated | 0.25 x 2.5 cm | Yes |
|  | 8 | Metal staples (edged, large) | 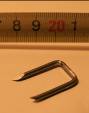 | Gardner Bender^®^, WI, USA | Recessed head, pointed legs and sharpened tips | 2.5 x 2.5 x 1.8 cm | Yes |
|  | 9 | Metal staples (rounded, large) | 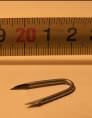 | Silvan, Denmark | Zinc coated, rounded head, pointed legs and sharpened tips | 2 x 2.5 cm | Yes |
|  | 10 | Metal staples (rounded, medium) | 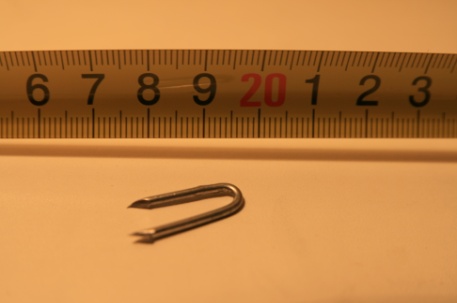 | Silvan, Denmark | Zinc coated, rounded head, pointed legs and sharpened tips | 1.6 x 2 cm | Yes |
|  | 11 | Metal staples (rounded, small) | 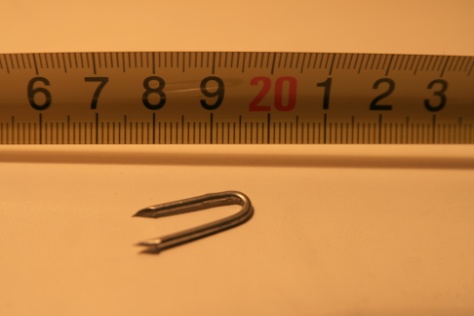 | Silvan, Denmark | Zinc coated, rounded head, pointed legs and sharpened tips | 1.6 x 1.5 cm | Yes |
|  | 12 | Metal staples | 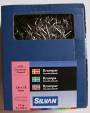 | Silvan, Denmark | Zinc coated, rounded head, pointed legs and sharpened tips | 1.6 x 1.5 cm | No |
|  | 13 | Nails | 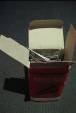 | Medina market, Bamako, Mali | Locally used for mud walls | 2.5 x 5 cm | Yes |
|  | 14 | Nails | 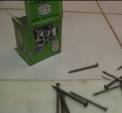 | Medina market, Bamako, Mali | Locally used for mud walls | 2.5 x 5 cm | No |
|  | 15 | Concrete nails | 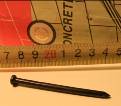 | Obuasi Central market, Ghana | Electrogalvanized nail with smooth shank and round head | 0.4 x 6.5 cm | No |
|  | 16 | Steel nails | 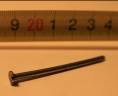 | Obuasi Central market, Ghana | Locally used for mud walls | 2.5 x 5 cm | Yes |
|  | 17 | Screw eyelet | 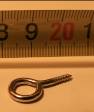 | Obuasi Central market, Ghana | Sealed hook | 2.5 cm | No |
|  | 18 | Screw hooks | 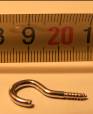 | Obuasi Central market, Ghana | Open hook | 2.5 cm | No |
|  | 19 | Screw hooks | 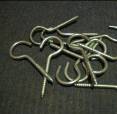 | Medina market, Bamako, Mali | Open hook | 4 cm | No |
|  | 20 | Wood fasteners | 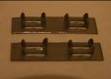 | Easy Gardener Products^®^, TX, USA | Galvanized metal | 5 x 1.5 cm | No |
|  | 21 | Non-metallic cable plastic staples | 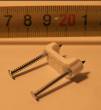 | Gardner Bender^®^, WI, USA | Zinc coated nails with recessed heads. UV-resistant polyethylene saddles | 12.7 cm | Yes |
|  | 22 | Cable plastic staples | 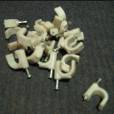 | Medina market, Bamako, Mali | Zinc coated nails | 1 cm | No |
| Adhesives: tapes | 23 | Double-sided carpet tape | 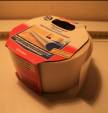 | TESA^®^, Germany | Transparent, double-sided | 5 m x 5 cm | Yes |
|  | 24 | Double-sided carpet tape | 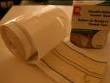 | Roberts^®^ Consolidated, FL, USA | Transparent, double-sided | 4.5 m x 6.5 cm | Yes |
|  | 25 | Outdoor mounting tape | 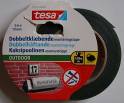 | TESA^®^, Germany | Dark green, single-sided | 5 m x 1.9 cm | Yes |
|  | 26 | Outdoor mounting tape | 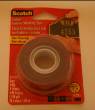 | 3M/Scotch^®^, USA | Red, single-sided | 1.52 m x 2.5 cm | Yes |
|  | 27 | PowerGrab heavy duty adhesive | 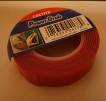 | Henkel Professional Adhesives, OH, USA | Red, double-sided | 1.5 m x 1.9 cm | Yes |
|  | 28 | Double-sided universal tape | 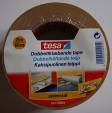 | TESA^®^, Germany | Beige, double-sided | 25 m x 5 cm | Yes |
|  | 29 | Double-sided tape | 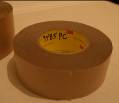 | 3M/Scotch^®^, USA | Beige, double-sided | 55 m x 5 cm | Yes |
|  | 30 | Double-sided tape | 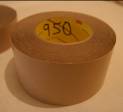 | 3M/Scotch^®^, USA | Beige, double-sided | 55 m x 7.6 cm | Yes |
|  | 31 | Double-sided tape | 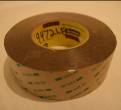 | 3M/Scotch^®^, USA | Beige, double-sided | 55 m x 5 cm | Yes |
|  | 32 | Power tape | 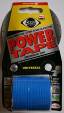 | Henkel, Denmark | Blue, single-sided | 5 m x 5 cm | Yes |
| Adhesives: glues | 33 | Fortissimo MS glue | 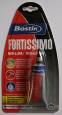 | Bostik, Denmark | MS-polymer based adhesive | 30 g | Yes |
|  | 34 | Ponal paper glue | 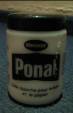 | Henkel, Medina market, Bamako, Mali | Locally used on wood | 200 g | Yes |
|  | 35 | Durabond | 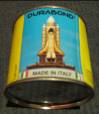 | Industrie Chimiche Forestali, Medina market, Bamako, Mali | High viscosity adhesive | 250 g | Yes |
|  | 36 | PowerGrab | 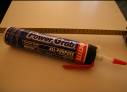 | Henkel, OH, USA | All-purpose construction adhesive | 0.2 L | Yes |
|  | 37 | Liquid Nails^®^ (LN-700) | 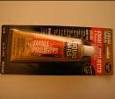 | PPG Architectural Coatings,  OH, USA | All-purpose construction adhesive | 0.7 L | Yes |
|  | 38 | DAP^®^ Weldwood^®^ Contact Cement | 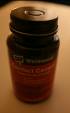 | DAP Products Inc., MD, USA | Neoprene based adhesive | 0.089 L | Yes |
|  | 39 | Stick-ease wall covering seam repair | 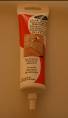 | Roman Decorating Products, IL, USA | Synthetic polymer based adhesive | 0.085 L | Yes |
|  | 40 | Border paste | 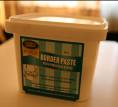 | Roman Decorating Products, IL, USA | Synthetic polymer based wall paper adhesive | 0.946 L | Yes |
|  | 41 | Golden Harvest TEKNAbond^®^ Multi-Purpose Wall Size/Adhesive | 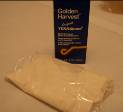 | Roman Decorating Products, IL, USA | All-purpose wall paper adhesive | 227 g | Yes |
|  | 42 | DAP^®^ Blue STIK^TM^ reusable adhesive putty | 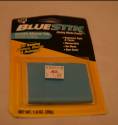 | DAP Products Inc., MD, USA | Blue adhesive putty | 28 g | Yes |
|  | 43 | AquaMend^®^ underwater repair epoxy putty | 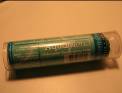 | Polymeric Systems Inc., PA, USA | Fibreglass-reinforced polymer | 57 g | Yes |
|  | 44 | Mud | 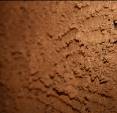 | Anwona, Ghana | Red mud | N/A | Yes |
| Materials | 45 | Multi-Purpose Ties | 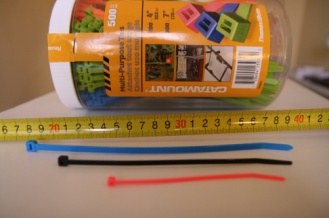 | Thomas and Betts Corporation, TN, USA | Blue, green and orange | 17.8 cm | No |
|  | 46 | Multi-Purpose Ties | 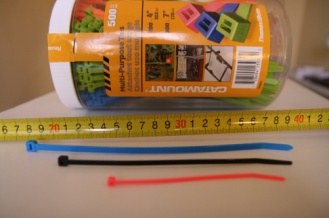 | Thomas and Betts Corporation, TN, USA | Black | 15.5 cm | No |
|  | 47 | Multi-Purpose Ties | 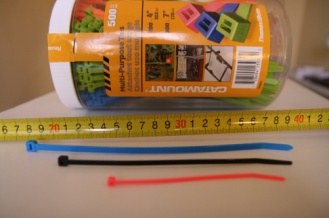 | Thomas and Betts Corporation, TN, USA | Yellow and pink | 10.2 cm | No |
|  | 48 | Shade net hooks | 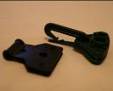 | Unknown source, USA | Black plastic hooks | N/A | No |
|  | 49 | Shower curtain rings | 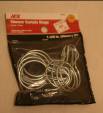 | Ace Hardware Corporation, IL, USA | Metal shower curtain rings | 3.5 cm | No |
|  | 50 | EZ Grabbit premium tarp tie down | 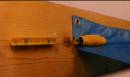 | Grabbit Tool Company, CO, USA | Yellow | 15 cm | No |
|  | 51 | CinchTite tarp zipper | 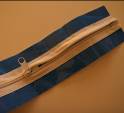 | Homax^®^ Products Inc., WA, USA | Reinforced white zipper | 0.1 x 2.1 m | No |
|  | 52 | Cord | 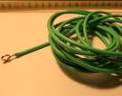 | Obuasi Central market, Ghana | Green PVC with a metal wire inside | 3 m | No |
|  | 53 | Cord | 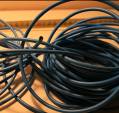 | Obuasi Central market, Ghana | Blue PVC with a metal wire inside | 30 m | No |
|  | 54 | Thin rope | 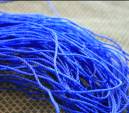 | Medina market, Bamako, Mali | Blue nylon | 20 m | No |
|  | 55 | Suspend-It^®^ light duty hanger wire install kit | 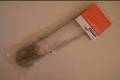 | Suspend-It^®^, FL, USA | 18-gauge hanger wire with eye lag screws | 0.8 m | No |
