# Supplementary material for: Installation of insecticide-treated durable wall lining: evaluation of attachment materials and product durability under field conditions
Source: Parasit Vectors. 2014 Nov 18;7:508. doi: 10.1186/s13071-014-0508-4 (PMC4246572; doi:10.1186/s13071-014-0508-4)
Supplement: Additional file 2: — Table S2. Specifications of sixteen potential durable wall lining fixing products evaluated during phase 2. [file 13071_2014_508_MOESM2_ESM.docx]

Supplementary Table 2. Specifications of sixteen potential durable wall lining fixing products evaluated during phase 2.

| **Type of fixing** | **Product Number #** | **Product** | **Product Image** | **Manufacturer, Source** | **Description** | **Size** |
| --- | --- | --- | --- | --- | --- | --- |
| Mechanical | 3 | Roofing nail (small) with Grip Rite^®^ plastic cap | 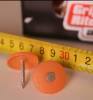 | Grip Rite Prime Source^®^ Building products Inc., TX, USA | Stainless steel nail with orange plastic cap covering nail head | 0.2 x 2.54 cm  (2.4 cm cap diameter) |
|  | 3b | Roofing nail (medium) with Grip Rite^®^ plastic cap | 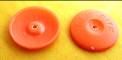 | Grip Rite Prime Source^®^ Building products Inc., TX, USA | Stainless steel nail with orange plastic cap covering nail head | 0.2 x 3.8 cm  (2.4 cm cap diameter) |
|  | 3c | Roofing nail (large) with Grip Rite^®^ plastic cap | 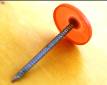 | Grip Rite Prime Source^®^ Building products Inc., TX, USA | Stainless steel nail with orange plastic cap covering nail head | 0.2 x 4.5 cm  (2.4 cm cap diameter) |
|  | 8 | Metal staples (edged, large) | 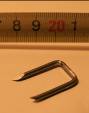 | Gardner Bender^®^, WI, USA | Recessed head, pointed legs and sharpened tips | 2.5 x 2.5 x 1.8 cm |
|  | 56 | Marked nail with Bostitch^®^ plastic cap | 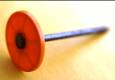 | Stanley Fastening Systems, LP., RI, USA | Local nail with orange plastic cap covering nail head | 0.3 x 5.1 cm  (2.5 cm cap diameter) |
|  | 57 | Fluted shank masonry with Bostitch^®^ plastic cap | 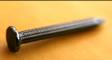 | Stanley Fastening Systems, LP., RI, USA | Galvanized nail with fluted shank for masonry | 0.4 x 3.8 cm  (2.5 cm cap diameter) |
|  | 58 | Fasteners | 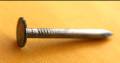 | Grip Rite Prime Source^®^ Building products Inc., TX, USA | Electrogalvanized roofing nail | 0.3 x 3.2 cm |
|  | 59 | Spiral shank nail with Bostitch^®^ plastic cap | 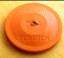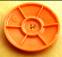 | Stanley Fastening Systems, LP., RI, USA | Stainless steel nail with orange plastic cap covering nail head | 0.28 x 5.1 cm  (2.5 cm cap diameter) |
|  | 60 | Marked nail | 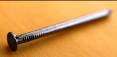 | Obuasi market, unknown manufacturer | All-purpose steel nail | 0.3 x 5.1 cm |
|  | 61 | Roofing nail #3 with Bostitch^®^ plastic cap | 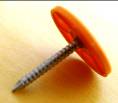 | Stanley Fastening Systems, LP., RI, USA | Stainless steel nail with orange plastic cap covering nail head | 0.2 x 2.54 cm  (2.5 cm cap diameter) |
|  | 62 | Spiral shank nail #59 with Grip Rite^®^ plastic cap | 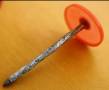 | Grip Rite Prime Source^®^ Building products Inc., TX, USA | Stainless steel nail with orange plastic cap covering nail head | 0.28 x 5.1 cm  (2.4 cm cap diameter) |
|  | 63 | Roofing nail from #3b with Bostitch^®^ plastic cap | 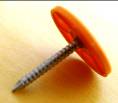 | Stanley Fastening Systems, LP., RI, USA | Stainless steel nail with orange plastic cap covering nail head | 0.2 x 3.8 cm  (2.5 cm cap diameter) |
| Adhesives: glues | 36 | PowerGrab | 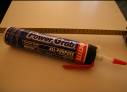 | Henkel, OH, USA | All-purpose construction adhesive | 0.2 L |
|  | 37 | Liquid Nails^®^ (LN-700) | 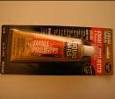 | PPG Architectural Coatings,  OH, USA | All-purpose construction adhesive | 0.7 L |
|  | 67 | 3M Spray | 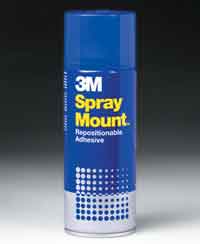 | 3M/Scotch^®^, USA | Aerosol spray adhesive | 0.7 L |
|  | 68 | Liquid Nails^®^ (LN-701) | 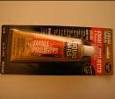 | PPG Architectural Coatings,  OH, USA | All-purpose construction adhesive | 0.7 L |
