# Supplementary material for: Installation of insecticide-treated durable wall lining: evaluation of attachment materials and product durability under field conditions
Source: Parasit Vectors. 2014 Nov 18;7:508. doi: 10.1186/s13071-014-0508-4 (PMC4246572; doi:10.1186/s13071-014-0508-4)
Supplement: Additional file 3: — File S1. Durable wall lining household installation protocol. [file 13071_2014_508_MOESM3_ESM.docx]

***Durable Wall Lining Installation Tools and Household Preparation***

ZeroVector^®^ DL (Vestergaard Frandsen, Switzerland) is commercially manufactured as rolls measuring 2.3 x 100 m, which are cut by an installation team to cover each house’s specific dimensions. The installation process can be divided into six stages and is carried out by a three person team, who are responsible for a separate task at each step (Table 1 and Figure 1). In general, person one installs the upper edge, or large rib, person two the lower large rib and person three handles the roll of DL. Members of the installation team are all equipped with personal protective equipment (long sleeves, long trousers, cotton caps, gloves and safety goggles) to prevent prolonged chemical exposure and a utility belt to hold equipment. In addition to the DL rolls, the following other tools are required: a measuring tape (5 m), cutting tool, two hammers, nails with Bostitch^®^ plastic nail caps, light source (torch and/or head lamps) and a ladder.

Householders should be notified 24 hours in advance to allow time for them to move their furniture into the centre of the room, leaving a 0.5 - 1 m gap of free space around the walls. All wall items, including, clothes, nails, pictures, posters and curtains must also be taken down. Windows are opened to allow in light and airflow. Residents can be present inside the house during the installation if they have any concerns about leaving the team unsupervised and/or if they wish to assist.

***Durable Wall Lining Installation Procedure***

Prior to DL installation, the perimeter of each inner house wall is measured. Outside the house, the DL is rolled out to fit the total dimensions and an extra 0.5 - 1 m is added to ensure there is sufficient material. The DL is cut, rolled, dusted off and labeled with a specific house identification number. If the DL cannot be measured outside, (e.g. during heavy rain) a smaller roll of DL (maximum 50 m) can instead be maneuvered by person three inside the house during the installation.

Installation begins in one corner, by nailing the DL to the top of the wall and by also fixing a 20 cm overlap onto an adjacent wall. The team work around the room (in the direction the DL roll is oriented), fixing the material to the walls with nails at 60 - 70 cm intervals (approximately three hand spans) placed in the upper and lower larger ribs. Person one and two fix the upper and lower large ribs at the same wall position simultaneously in order to stretch and attach the DL evenly (person three is responsible for coordinating the DL roll). The DL must be fixed at least 3 cm above the ground and not folded, to prevent dust or mold gathering. This is achieved by person two continuously pulling down on the fabric while person one holds the upper large rib in position. Person three ensures the lining is stretched and aligned neatly, with nails placed at the same distance in both ribs.

It is important to place the DL as high as possible to cover the majority of the wall. In houses where the DL is wider than the wall height (more than 2.3 m), it can either be attached to roofing beams or the ceiling (using one nail on the wall and one on the ceiling) or rolled inwards, using the upper rib as the rolling height. If the DL is less wide than the wall height it should be aligned to top of the wall and not floor level. In this case, two rolls of DL can be used to cover the whole wall and fixings should be placed in the middle of the large rib and through all folded layers, where necessary.

During the installation, each attachment point is evaluated for grip and if there are any noticeable failures then the fixing is moved along the large rib and/or attached to alternative materials, such as wooden beams. Every corner will initially be fixed by a nail and cap in the upper and lower large ribs. However, in houses with mud walls, the corners tend to be weaker and fixings may also need to be placed on the adjacent walls for additional support.

When reaching the last corner there should be excess material which is cut to cover the initial 20 cm overlap. The remaining DL material is folded lengthwise by 20 cm and fixed over the 20 cm overlap in the upper and lower large ribs.

To cut a hole for the door, a vertical slit is made 15cm into the door space, to allow the DL to shrink and tilted cuts are made in all upper and lower corners. To seal these vertical cuts, each overlapping material is folded by 15 cm and fixed to the door frame or the adjacent walls.

When the door can be opened, the house owners are invited inside and asked to decide whether they would like the DL to screen the windows and/or eaves or for them to be cut open. If the residents prefer screening, four to five nails can be used to fix the DL around the window. If the windows are to be opened then they are sealed in a similar manner to the door (tilted cuts, 15 cm overlap and fixed using nails).

After the DL is attached in the top and bottom ribs and around the windows and door, then the remaining fixings can be used to reinforce the DL in the middle sections, where necessary. In general, for a house with a 15 m perimeter, a maximum of 100 nails are needed (Figure 2).

Following installation, person three is responsible for informing house owners of safety precautions and how to maintain the DL, including to clean it with a brush or a wash cloth and not to paint or remove it or burn mosquito coils, incense or candles close to the walls.

***Local Adaptation of Durable Wall Lining Installation***

In situations where wall surfaces are too hard, causing nails to bend easily, grip of nail should be evaluated and the cap sealed to the DL by bending the nail head. In houses where the wall surface has a very limited grip, for example, a thin layer of concrete with corroded mud rendering, a number of fixings may be needed and the house owners may have to move the fixings when the nail grip fails.

When houses have thatch, bamboo, palm leaves or hay as roofing, another method may be required to fix the top of the DL. In these cases, plastic cable ties or string can be purchased at a local market and used to tie the upper large rib onto the roofing fibres. This also screens open eaves that are often present in houses with this type of roofing. Alternatively, if wooden beams used for the roof are stable and strong then nails with caps can be fixed to these. In some cases external wooden support beams have been reported to be difficult to locate from within a house, necessitating a fourth person to participate in the installation by standing outside and directing the placement of fixings [1].

In some communities, cultural furnishings may need to be considered and the installation procedure adapted accordingly. For example, in parts of Vietnam designated electrical points and alters are commonly found on livings room walls, which must remain uncovered [2] and in Papua New Guinea, traditional homes have bed stands and tables embedded into dirt floors, pressed against interior walls, preventing DL installation on these surfaces [1].

Table 1. Durable Wall Lining Installation Protocol

| **Procedure Stage** | **Person 1** | **Person 2** | **Person 3** | **Tools Required** |
| --- | --- | --- | --- | --- |
| **1. Preparation** | Roll out DL outside house, measure, cut and roll  Add 0.5 - 1 m length | Assist Person 1 | Ensure house is prepared, i.e. remove items on or along the wall, curtains etc.  Put caps on nails | Cutting tool  Measuring tape  Pens |
| **2. Wall Fixing** | Hammer nails in top rib at 60 - 70 cm distance  Nice folds using rib if walls are low  Fix DL as high as possible | Hammer nails in bottom rib at 60 - 70 cm distance  DL must not touch the ground (3 cm above)  No folding  Follow height of DL to be defined by Person 1  Follow fixing speed of person 1 | Hold DL roll  Roll out fabric when necessary  Ensure person 1 and 2 are working at the same spend and textile is aligned and stretched evenly | 2 hammers  3 caps  3 pairs of gloves, tool/nails belts  Ladder to stand on  100 nails with caps |
| **3. Cut Door** | Hold DL for cutting | Cut 15 cm overlap | Assist if necessary | Cutting tool |
| **4. Fix At Door** | Fold 15 cm overlap and fix  Align nicely to door frame | Fold 15 cm overlap and fix  Align nicely to door frame | Consult house owner if they want to have open windows or fixed screening | 2 hammers  Nails with caps |
| **5. Window** | Fix/cut around window | Fix/cut around window | Inform house owner of how folds can be reinforced and how to clean and care for the DL installation | 2 hammers  Cutting tool  Nails with caps |
| **6. Finish** | Fix rest of nails – first corners then in middle where necessary  Maximum 100 nails for 3 x 4 m house  Clean the DL with a damp cloth if it is dirty | Collect tools/ladder/chair etc. | Go to next house and ensure it is ready for installation, i.e. remove items on or along the wall, curtains etc. | 2 hammers  Nails with caps |


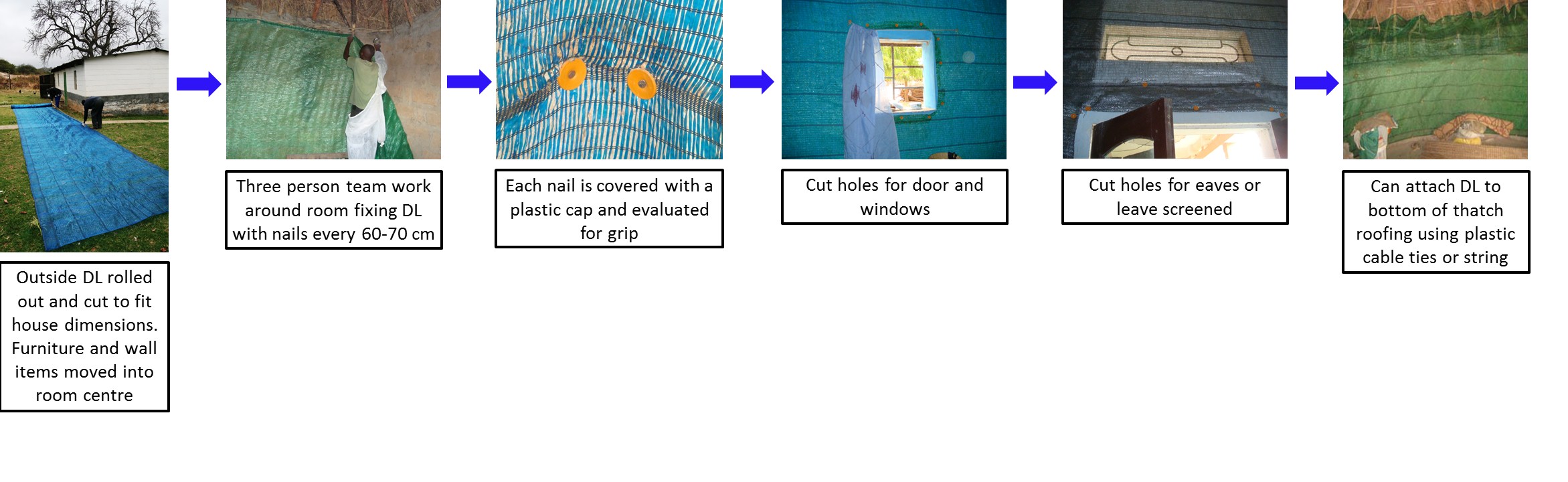


Figure 1. Stages of durable wall lining installation.


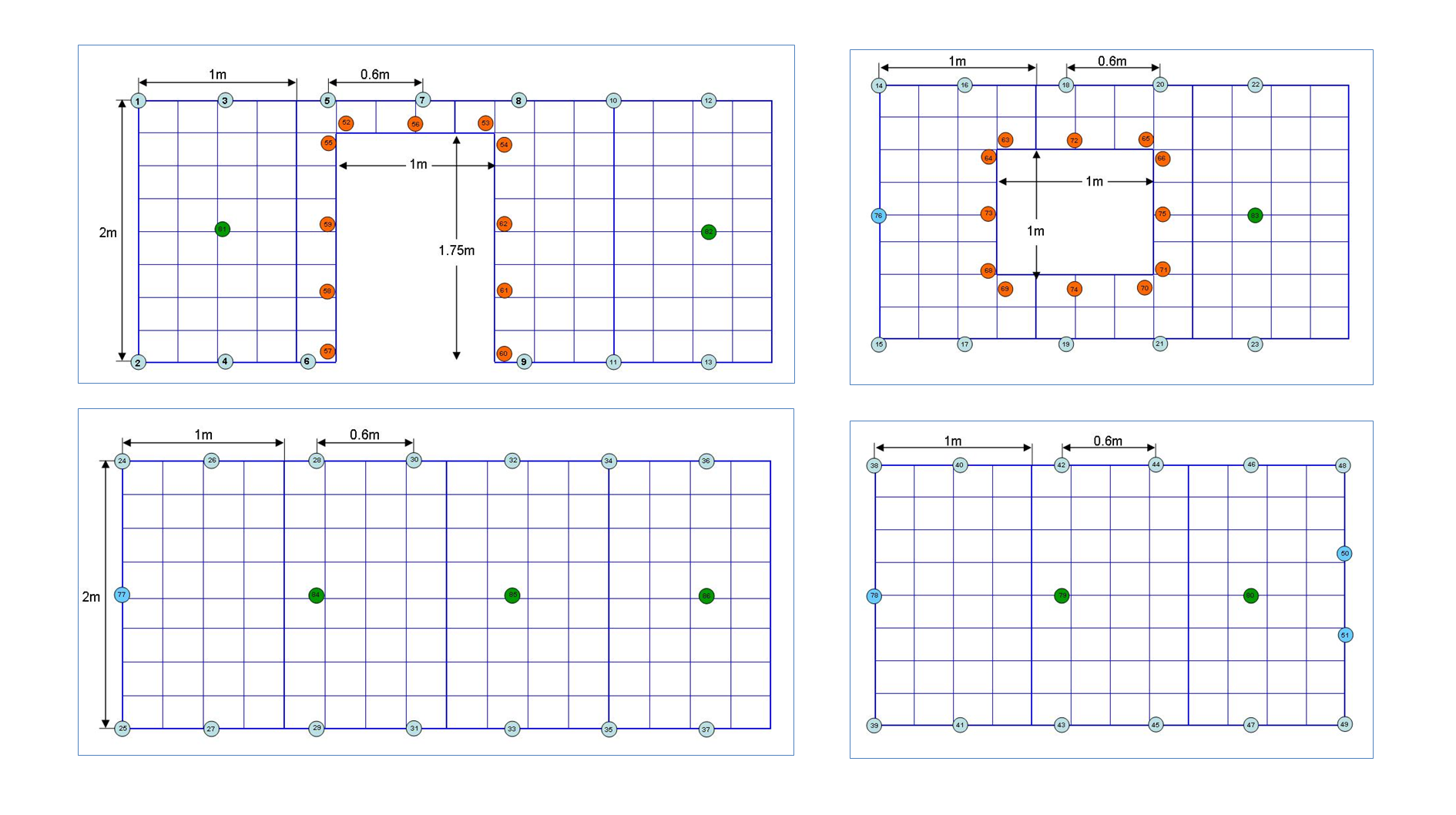


Figure 2. Template for a 3 x 4 m house unit with a 1 x 1.75 m door and one 1 x 1 m window. The total number of nails used is 86. Colours correspond to the order of fixings. Light blue are initial fixings, orange are window and door fixings, turquoise are corners and green are finishing fixings.

***References***

1. Pulford J, Tandrapah A, Atkinson JA, Kaupa B, Russell T, Hetzel MW: **Feasibility and acceptability of insecticide-treated plastic sheeting (ITPS) for vector control in Papua New Guinea.** *Malar J* 2012, **11:** 342.
2. Messenger LA, Matias A, Manana AN, Stiles-Ocran JB, Knowles S, Boakye DA, Coulibaly MB, Larsen ML, Traoré AS, Diallo B, Konaté M, Guindo A, Traoré SF, Mulder CEG, Le H, Kleinschmidt I, Rowland M: **Multicentre studies of insecticide-treated durable wall lining in Africa and South-East Asia: entomological efficacy and household acceptability during one year of field use.**  *Malar J* 2012, **11:** 358.
